# Proteomic Profile in Retinopathy of Prematurity: A Secondary Analysis of the Mega Donna Mega Randomized Clinical Trial

**DOI:** 10.1001/jamaophthalmol.2025.5594

**Published:** 2026-01-08

**Authors:** Pia Lundgren, Hanna Danielsson, Mohit B. Panwar, María Bueno Álvez, Aldina Pivodic, Wen Zhong, Nele Brusselaers, Dirk Wackernagel, Ulrika Sjöbom, Karin Sävman, Ingrid Hansen Pupp, David Ley, Susanna Klevebro, Anders K. Nilsson, Zhongjie Fu, Lois E. H. Smith, Mathias Uhlén, Ann Hellström

**Affiliations:** 1 The Sahlgrenska Centre for Pediatric Ophthalmology Research, Department of Clinical Neuroscience, Institute of Neuroscience and Physiology, Sahlgrenska Academy, University of Gothenburg, Gothenburg, Sweden; 2 Department of Ophthalmology, Sahlgrenska University Hospital, Region Västra Götaland, Gothenburg, Sweden; 3 Department of Women’s and Children’s Health, Karolinska Institutet, Stockholm, Sweden; 4 Centre for Translational Microbiome Research, Department of Microbiology, Tumor and Cell Biology, Karolinska Institutet, Stockholm, Sweden; 5 Sach’s Children’s and Youth Hospital, Södersjukhuset, Stockholm, Sweden; 6 Science for Life Laboratory, Department of Protein Science, KTH Royal Institute of Technology, Stockholm, Sweden; 7 University of Gothenburg Centre for Person-Centered Care, Sahlgrenska Academy, University of Gothenburg, Sweden; 8 Science for Life Laboratory, Department of Biomedical and Clinical Sciences (BKV), Linköping University, Linköping, Sweden; 9 Department of Neuroscience, Karolinska Institutet, Stockholm, Sweden; 10 Science for Life Laboratory, Department of Protein Science, KTH—Royal Institute of Technology, Stockholm, Sweden; 11 Global Health Institute, Department of Family Medicine and Population Health, University of Antwerp, Antwerp, Belgium; 12 Division of Neonatology, Department of Pediatrics, University Medical Center of the Johannes Gutenberg-University Mainz, Mainz, Germany; 13 Department of Clinical Neuroscience, Institute of Neuroscience and Physiology, Sahlgrenska Academy, University of Gothenburg, Gothenburg, Sweden; 14 Department of Pediatrics, Institute of Clinical Sciences, Sahlgrenska Academy, University of Gothenburg, Gothenburg, Sweden; 15 Region Västra Götaland, Department of Neonatology, The Queen Silvia Children´s Hospital, Sahlgrenska University Hospital, Gothenburg, Sweden; 16 Paediatrics, Department of Clinical Sciences, Lund University, Lund, Sweden; 17 Department of Neonatology, Skåne University Hospital, Lund, Sweden; 18 Department of Clinical Science and Education, Karolinska Institutet, Södersjukhuset, Stockholm, Sweden; 19 The Department of Ophthalmology, Boston Children’s Hospital, Harvard Medical School, Boston, Massachusetts

## Abstract

**Importance:**

Identifying early proteomic profiles in infants who develop severe retinopathy of prematurity (ROP) may reveal targets for preventive interventions to reduce retinal vessel loss and the subsequent risk of severe ROP.

**Objective:**

To assess early longitudinal profiles of blood protein levels in preterm infants with or without severe ROP and the effect of arachidonic acid (AA) and docosahexaenoic acid (DHA) supplementation.

**Design, Setting, and Participants:**

This was an exploratory, post hoc analysis of serum proteome profiles in preterm infants in the double-masked Mega Donna Mega (MDM) randomized clinical trial using targeted Olink Proximity Extension Assay proteomics covering 538 analytes. The setting was 3 university hospitals in Sweden and included extremely preterm infants born before 28 weeks of gestational age (GA), from 2016 to 2019. Data were analyzed from January to March 2025.

**Exposures:**

All infants received standard nutrition; additionally, half received enteral lipid supplementation with AA/DHA (100/50 mg/kg per day) from birth to term equivalent age.

**Main Outcomes and Measures:**

Longitudinal protein profiles during the first month of life were examined using mixed models for repeated measures, adjusted for GA, study center, and AA/DHA supplementation, and tested for the interaction between severe ROP (stage ≥3 and/or treated) and postnatal age.

**Results:**

A total of 177 extremely preterm infants (mean [SD] GA, 25.6 [1.4] weeks; 100 male [56.5%]) were included, of whom 50 (28.2%) developed severe ROP. Of 538 longitudinal analyzed proteins, 109 protein profiles in the first month of life associated with severe ROP, proteins related to immune response, apoptotic processes, blood coagulation, and lipid metabolism. The most pronounced association with severe ROP was a fast rise in fibroblast growth factor 21 (FGF-21; β = 0.68; 95% CI, 0.39-0.97; *Q *=.002) and tissue plasminogen activator (tPA; β = 0.21; 95% CI, 0.13-0.29; *Q *<.001) during the first postnatal days. The increase in serum FGF-21 level in the first week of life was associated with lower GA, lower birth weight, low enteral energy intake, and more days receiving mechanical ventilation. No association was observed between AA/DHA supplementation and the proteome.

**Conclusions and Relevance:**

In this post hoc exploratory analysis of data from the MDM randomized clinical trial, a fast rise in FGF-21 levels, a metabolic stress-induced hormone, during the first postnatal days was strongly associated with the development of severe ROP in extremely preterm infants. These findings suggest that early interventions improving bioenergetic status may help prevent severe ROP.

**Trial Registration:**

ClinicalTrials.gov Identifier: NCT03201588

## Key Points

**Question:**

Are early longitudinal changes in proteomic profiles in extremely preterm infants associated with the development of severe retinopathy of prematurity (ROP)?

**Findings:**

In this secondary analysis of the Mega Donna Mega (MDM) randomized clinical trial including 177 extremely preterm infants, a faster rise in blood levels of the metabolic stress-induced protein fibroblast growth factor 21 (FGF-21) in the first postnatal days distinguished extremely preterm infants who later developed severe ROP. FGF-21 levels were associated with the infant’s degree of immaturity at birth, low enteral energy intake, and days receiving mechanical ventilation.

**Meaning:**

This study found that an early fast postnatal rise in FGF-21 levels may reflect metabolic and environmental stress influencing ROP pathogenesis, and improving bioenergetics may help prevent severe ROP.

## Teaser

This secondary analysis of the Mega Donna Mega (MDM) randomized clinical trial investigated if early longitudinal changes in proteomic profiles in extremely preterm infants are associated with the development of severe retinopathy of prematurity.

## Introduction

Infants born extremely preterm who develop severe retinopathy of prematurity (ROP), stage 3 or higher and/or treated are at high risk of visual impairment.^1^ ROP development is strongly influenced by the infant’s degree of prematurity, but suboptimal nutritional support as well as high or fluctuating oxygen levels, neonatal complications, and poor postnatal growth are additional risk factors.^2^

ROP is a 2-phase disease. Metabolic changes and environmental stress in the infants’ first weeks of life may cause retinal vessel loss (phase I ROP), potentially leading to proliferative severe ROP diagnosed weeks or months later (phase II ROP). After preterm birth, retinal vascularization is inhibited due to reduced levels of vasoactive growth factors such as vascular endothelial growth factor (VEGF) and insulinlike growth factor 1 (IGF-1), likely caused by hyperoxia and energy deficiency in the neuroretina (phase I ROP). Weeks later, increasing ischemia and inflammation in the avascular retina stimulate the increased production of growth factors such as VEGF, leading to disorganized retinal neovascularization (phase II ROP). If left untreated, neovascularization poses a risk for retinal detachment and vision loss.^3^

In a Swedish randomized clinical trial (RCT) including extremely preterm infants, the Mega Donna Mega (MDM) trial, additional enteral postnatal supplementation with arachidonic acid (AA) and docosahexaenoic acid (DHA) compared with standard nutrition reduced the incidence of severe ROP by 50%.^4,5^ Increased levels of AA and DHA, regardless of supplementation, were associated with reduced inflammatory responses and improved platelet function.^6,7^

Recent advancements in proteomics have enabled the simultaneous analysis of hundreds to thousands of proteins from a small blood sample, greatly facilitating longitudinal studies in preterm infants.^8^

In this post hoc exploratory analysis of the MDM trial, we examined longitudinal blood protein profiles and their association with severe ROP. We hypothesized that early changes in protein levels could help identify high-risk infants for severe ROP.

## Methods

### Participants

The study cohort consisted of infants born before 28 weeks of gestational age (GA) born at 3 university hospitals in Sweden between 2016 and 2019, participating in the Swedish MDM RCT. Half of the infants received enteral supplementation with AA/DHA (100/50 mg/kg per day) in addition to standard nutrition, from birth until their term-equivalent age. The trial oral supplement consisted of a triglyceride oil (Formulaid [DSM Nutritional Products Inc]). The trial’s primary outcome was the effect of the supplementation on severe ROP (the trial protocol and statistical analysis plan are available in Supplement 1 and Supplement 2, respectively).^4,5^ Data concerning infant race and ethnicity were not gathered in this study as Sweden lacks standardized definitions and categories for race and ethnicity.

In this secondary study that was not prespecified, longitudinal blood protein levels were evaluated in association with ROP outcome. The Regional Ethics Review Board in Gothenburg and the Swedish Ethical Review Authority approved the trial protocol. The study was conducted following the principles of the Declaration of Helsinki and the Consolidated Standards of Reporting Trials (CONSORT) reporting guidelines. Written informed consent to participate was provided by the participants’ parents or legal guardians.

### Clinical Data Collection, ROP Examinations, and Definitions

Clinical data regarding birth characteristics, neonatal morbidities, and nutritional support were recorded and previously published.^4,5^ In this study, we additionally included chorioamnionitis, defined if at least 2 of the following criteria were present: maternal fever higher than 38 °C, maternal C-reactive protein (CRP) greater than 2.0 mg/dL (to convert to milligrams per liter, multiply by 10), foul-smelling amniotic fluid, persistent maternal tachycardia (>100 beats per minute) or fetal tachycardia (>160 beats per minute), or antibiotic treatment due mothers uterine infection. Laboratory values were retrieved from the infant’s medical record. We defined anemia as hemoglobin less than 11.0 g/dL (to convert to grams per liter, multiply by 10), thrombocytopenia as platelet count less than 100 ×10^3^/µL (to convert to ×10^9^/L, multiply by 1), and hyperglycemia as glucose greater than 180.18 mg/dL (to convert to millimoles per liter, multiply by 0.0555).

ROP classification and treatment recommendations followed international standards.^9,10^ We defined severe ROP as ROP stage 3 or greater and/or treated.

### Blood Sample Collection and Blood Protein Profiling

Blood samples for targeted proteomics were taken according to study protocol at postnatal age (PNA) days 0 to 1, 3, 7, 14, and 28, followed by postmenstrual age weeks 30, 32, 36, and 40, details published previously.^8^ In summary, protein analysis was performed using the proximity extension assay coupled to quantitative real-time polymerase chain reaction^11^ by Olink (Uppsala, Sweden). After removing duplicates and applying quality control procedures, 538 proteins remained of the original 552 proteins on 6, 92-marker panels (Cardiometabolic, version 3603; Cardiovascular II, version 5006; Cardiovascular III, version 6113; Development, version 3512; Metabolism, version 3402; and Inflammation, version 3022). Protein abundances are expressed in Normalized Protein eXpresion (NPX), an arbitrary unit on a log_2_ scale. A high NPX value corresponds to a high serum protein abundance. In this study, we focused on the infants’ protein profiles from birth to day 28 or the closest available day up to day 38.

### Statistical Analyses

Continuous variables were described by mean, SD, median, minimum, maximum, and IQR, and categorical variables by counts and percentages. For tests between 2 groups, Fisher exact test was used for dichotomous variables, the χ^2^ test for categorical variables, and the Mann-Whitney *U* test for continuous variables.

Principal component analysis (PCA) was used to explore major sources of variance in the protein levels over PNA. For trajectory analyses, cubic spline and piecewise linear mixed models (with fixed break points at tertiles: 4 and 15 PNA days) with random intercepts were used to examine associations between protein trajectories (as the dependent variable) and severe ROP (as the main fixed effect). Separate models were fitted for each of the 538 proteins. Similar models were applied when evaluating the effect of AA/DHA supplementation on the proteome in the first month of life. All models were adjusted for GA, study center, and randomized supplementation. To assess whether the association between severe ROP and protein levels varied over PNA, an interaction term between severe ROP and PNA was included. *Q* values were provided as adjusted *P* values following Benjamini-Hochberg false discovery rate (FDR) adjustment.

Protein-protein interaction networks were constructed using STRINGdb, version 2.21.0 with STRING database, version 12.0, for proteins showing severe ROP interactions (R Project for Statistical Computing). Louvain clustering (igraph, version 2.1.4, resolution = 2.5) identified functional clusters, with enrichment analysis performed for Gene Ontology, KEGG (Kyoto Encyclopedia of Genes and Genomes), and WikiPathways terms. Networks were visualized using ggraph, version 2.2.1, with Fruchterman-Reingold layout, where node sizes represented likelihood ratio test significance (−log_10_ FDR) and cluster boundaries were marked with colored ellipses (ggforce, version 0.4.2).

Functional enrichment analysis was performed using GSEApy, version 1.1.3, for proteins showing severe ROP interactions in piecewise model within each time frame.^12^ Overrepresentation analysis tested gene ontology terms with FDR less than 0.05. Results were visualized as horizontal bar plots with gene category proportions color-coded by interaction direction.

Explained variances for early changes in fibroblast growth factor 21 (FGF-21) and tissue plasminogen activator (tPA), expressed as *R*^2^, were derived from univariate linear regression models, considering GA, birth weight, sex, chorioamnionitis, and clinical variables on mechanical ventilation, energy intake (enteral and parenteral nutrition), maximum CRP, maximum glucose, minimum hemoglobin, minimum platelet counts, sepsis, and AA and DHA changes, during first postnatal week, in separate models. FGF-21, tPA, AA, and DHA were analyzed as slopes, representing their rate of change during the first postnatal week, derived from the individual linear regressions based on data from PNA days 0 to 10. Missing data was not imputed. All analyses were performed using SAS software, version 9.4 (SAS Institute Inc) and R, version 4.5.0 (R Project for Statistical Computing). All *P* values were calculated using 2-sided tests, and *P* <.05 or *Q* <.05 was considered statistically significant. Data were analyzed from January to March 2025.

## Results

### Clinical Characteristics of the Study Cohort

We evaluated 209 infants from the MDM cohort, of which 177 (mean [SD] GA, 25.6 [1.4] weeks; 77 female [43.5%]; 100 male [56.5%]) had an exact final ROP staging (eFigures 1 and 2 in Supplement 3).

Among the 177 infants included, 50 (28.2%) developed severe ROP, but less frequently in the AA/DHA supplemented, 16 of 84 (19.0%) vs 34 of 93 (36.6%) without AA/DHA supplemented (difference, 17.5%; 95% CI, 4.6%-30.4%; *P* = .01), as previously reported.^4^ Infants with severe ROP experienced more neonatal complications in their first month and received less enteral nutrition support (Table^13^).

**Table.** Infant Characteristics for Patients With and Without Severe Retinopathy of Prematurity (ROP)^a^

Variable	No. (%)		*P* value
	No severe ROP (no ROP, stage 1 and 2) (n = 127)	Severe ROP (stage ≥3 and/or treated) (n = 50)	
Birth characteristics			
Gestational age, mean (SD), wk	26.0 (1.4)	24.6 (1.2)	<.001
Birth weight, mean (SD), g	850.5 (203.6)	692.3 (138.1)	<.001
Birth weight SDS (Fenton), mean (SD)	0.15 (0.82)	−0.03 (0.81)	.21
Sex			
Female	60 (47.2)	17 (34.0)	.13
Male	67 (52.8)	33 (66.0)	
Center			
1	43 (33.9)	22 (44.0)	.049
2	34 (26.8)	18 (36.0)	
3	50 (39.4)	10 (20.0)	
Chorioamnionitis	51 (41.5)	12 (24.0)	.04
Neonatal morbidities			
Any BPD^b^	62 (49.6)	35 (70.0)	.02
NEC	6 (4.7)	8 (16.0)	.02
PDA treatment			<.001
No treatment	68 (55.3)	13 (26.0)	
Pharmacological treatment only	47 (38.2)	22 (44.0)	
Instrumental	8 (6.5)	15 (30.0)	
Severe IVH, (stages 3 and 4)	13 (10.2)	8 (16.0)	.31
Mechanical ventilation, <1 wk PNA, mean (SD)	2.7 (2.9)	5.1 (2.7)	<.001
Mechanical ventilation, <4 wk PNA, mean (SD)	8.3 (10.1)	18.1 (9.0)	<.001
Nutritional support			
Total energy intake enterally, <1 wk PNA, mean (SD), kcal/kg/d	326.2 (158.2)	230.9 (140.5)	<.001
Total energy intake enterally, <4 wk PNA, mean (SD), kcal//kg/d	2909.4 (882.7)	2104.3 (1073.5)	<.001
Total energy intake parenterally, <1 wk PNA, mean (SD), kcal/kg/d	334.6 (123.2)	410.4 (106.4)	<.001
Total energy intake parenterally, <4 wk PNA, mean (SD), kcal/kg/d	602.8 (456.1)	1132.0 (745.6)	<.001
Slope AA, <1 wk PNA, mean (SD)	−0.52 (0.35)	−0.61 (0.40)	.27
Slope DHA, <1 wk PNA, mean (SD)	−0.09 (0.10)	−0.08 (0.09)	.32
Blood parameters			
Min hemoglobin, <1 wk PNA, mean (SD), g/dL	11.90 (1.89)	10.76 (1.23)	<.001
Min hemoglobin, <4 wk PNA, mean (SD), g/dL	10.19 (1.73)	9.78 (1.25)	.04
Min platelet count, <1 wk PNA, (100 ×10^3^/µL), mean (SD)	185.1 (77.4)	142.4 (78.5)	.001
Min platelet count, <4 wk PNA, (100 ×10^3^/µL), mean (SD)	176.0 (80.0)	119.8 (76.2)	<.001
Max CRP, <1 wk PNA, mean (SD), mg/dL	0.52 (0.77)	1.26 (1.63)	<.001
Max CRP, <4 wk PNA, mean (SD), mg/dL	1.40 (2.50)	3.76 (4.70)	<.001
Max glucose, <1 wk PNA, mean (SD), mg/dL	241.44 (156.76)	326.13 (176.58)	<.001
Max glucose, <4 wk PNA, mean (SD), mg/dL	268.47 (169.37)	365.77 (192.79)	<.001
Sepsis, <1 wk PNA	2 (1.6)	2 (4.0)	.32
Sepsis, <4 wk PNA	12 (9.4)	10 (20.0)	.08
Anemia, <1 wk PNA	34 (26.8)	25 (50.0)	.004
Anemia, <4 wk PNA	97 (76.4)	44 (88.0)	.10
Thrombocytopenia, <1 wk PNA	14 (11.3)	15 (30.0)	.006
Thrombocytopenia, <4 wk PNA	20 (15.7)	21 (42.0)	<.001
Hyperglycemia, <1 wk PNA	71 (55.9)	41 (82.0)	.001
Hyperglycemia, <4 wk PNA	82 (64.6)	47 (94.0)	<.001

Abbreviations: AA, arachidonic acid; BPD, bronchopulmonary dysplasia, CRP, C-reactive protein; DHA, docosahexaenoic acid; IVH, intraventricular hemorrhage; max, maximum; min, minimum; NEC, necrotizing enterocolitis; PNA, postnatal age; SDS, standard deviation score.

SI conversion factors: To convert CRP to milligrams per liter, multiply by 10; glucose to millimoles per liter, multiply by 0.0555; platelets to ×10^9 ^per liter, multiply by 1, hemoglobin to grams per liter, multiply by 10.

^a^ For tests between 2 groups with respect to dichotomous variables Fisher exact test was used, for categorical variables χ^2^ test was used, and for continuous variables, Mann-Whitney *U* test was used.

^b^ No BPD vs BPD grade 1, 2, or 3 according to Jensen et al.^13^

### Study Design and Serum Proteome Over Time

**Figure 1.**
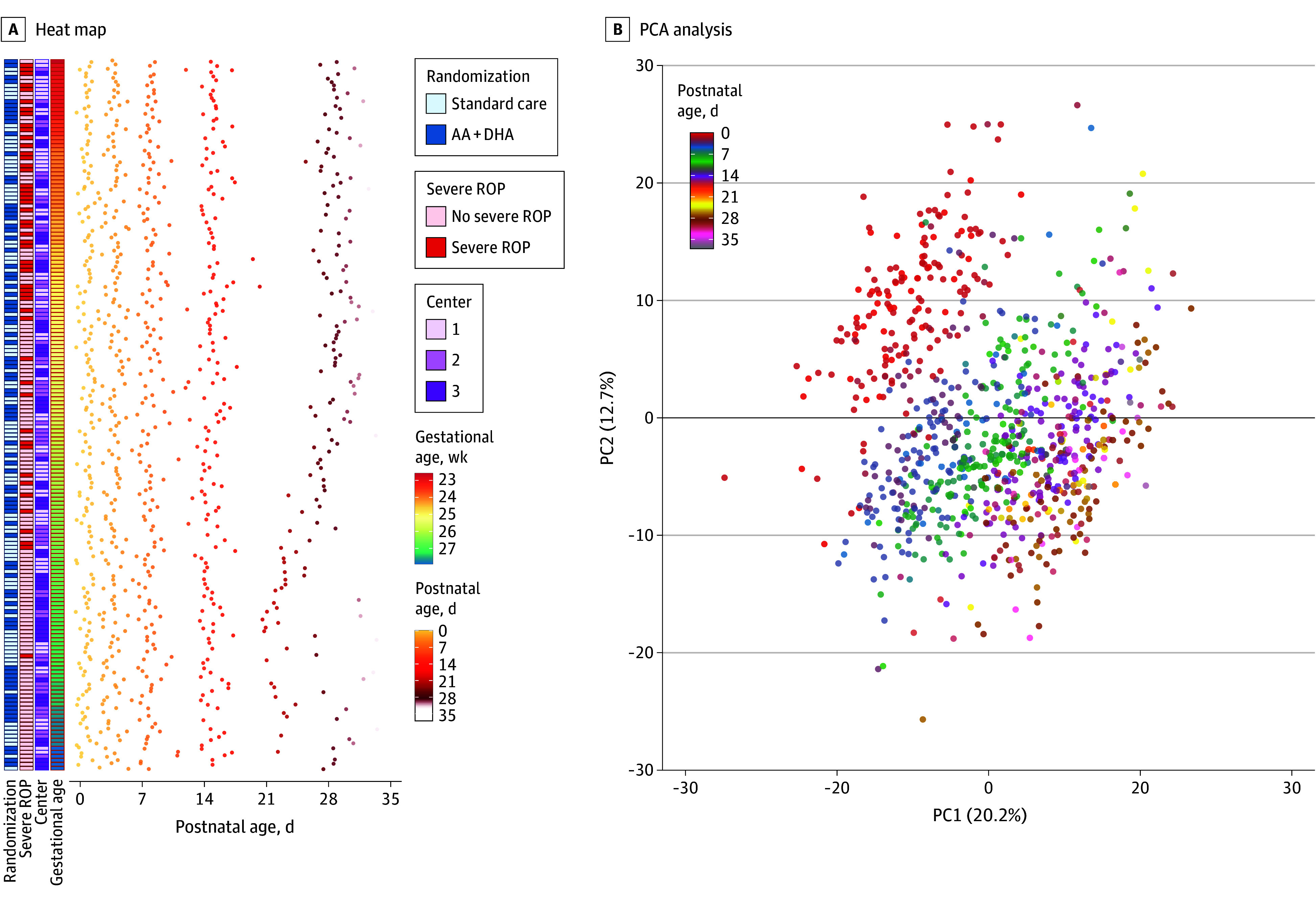
Overview of the Study A, Heat map of observations per infant sorted by gestational age at birth. Each dot represents one serum sample used for proteomics. B, Multilevel principal component analysis (PCA) showing postnatal changes in the serum proteome. Numbers in parentheses show % of total variance explained by principal component (PC) 1 (*x*) and PC2 (*y*). AA indicates arachidonic acid; DHA, docosahexaenoic acid; ROP, retinopathy of prematurity.

An overview of the study design and the postnatal development of the proteome is presented in eFigure 1 in Supplement 3 and Figure 1A, respectively. A clear PNA-dependent shift in the proteome was observed over the first month of life, as visualized by PCA (Figure 1B), as partly previously reported.^8^

### Neonatal Protein Profiles and Severe ROP

**Figure 2.**
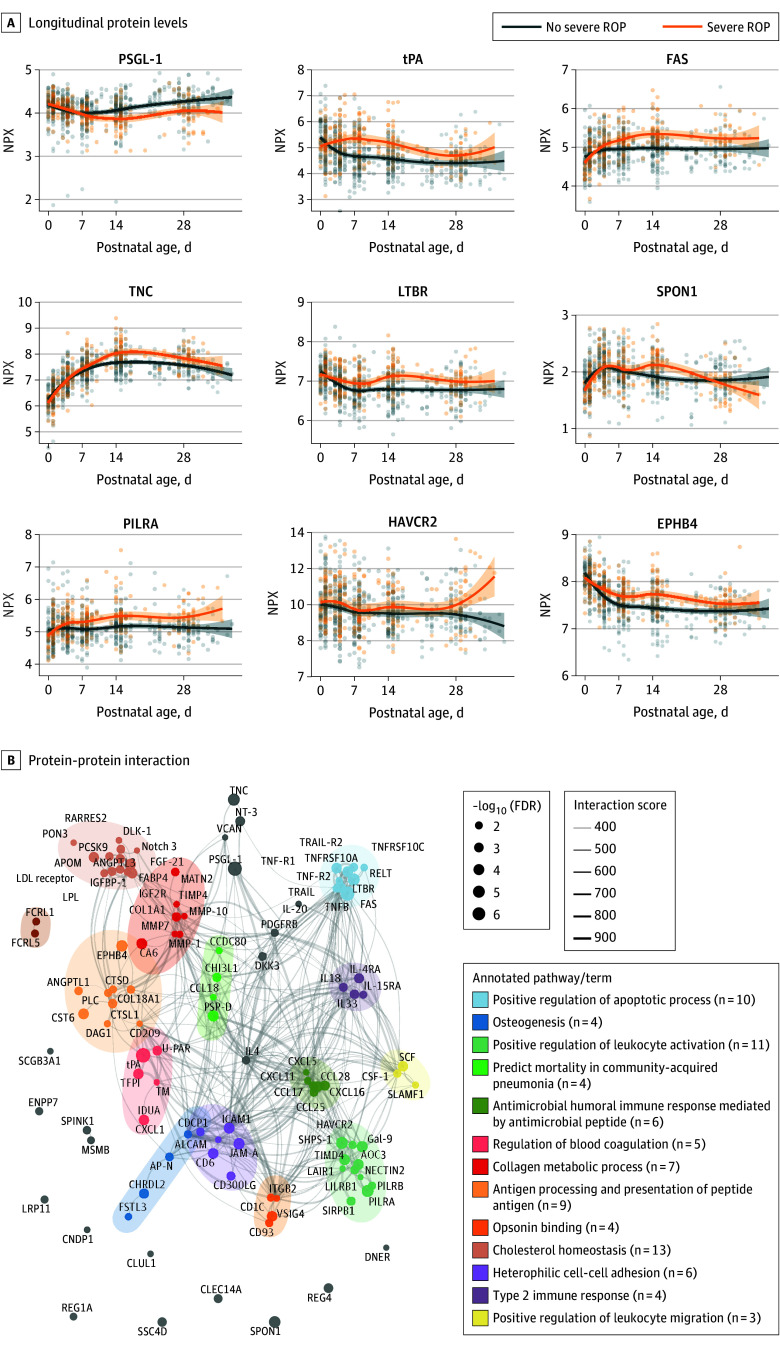
Longitudinal Protein Levels and Protein-Protein Interactions A, Longitudinal protein levels during first month of life according to final retinopathy of prematurity (ROP) stage, no severe ROP (stage 0, 1, and 2), or severe ROP (ROP stage 3 and/or treated ROP) for the top 9 proteins with the lowest *Q* values from cubic spline mixed models for repeated measures. B, Protein-protein interaction network using STRING (Search Tool for the Retrieval of Interacting Genes/Proteins) database based on the 109 proteins with an interaction between postnatal age and final ROP stage. Functional annotation of pathways/terms from Louvain clustering with enrichment analysis performed for Gene Ontology, KEGG (Kyoto Encyclopedia of Genes and Genomes), and WikiPathways terms are highlighted. Term expansions of all proteins are available in eAppendix 1 in Supplement 3.

After FDR, 109 proteins showed an interaction between severe ROP and PNA first month of life (eTable 1 in Supplement 4). Longitudinal protein levels according to no severe ROP or severe ROP for the top 9 proteins with the lowest *Q* values are presented in Figure 2A. The 3 proteins with the most prominent interaction between severe ROP and PNA were P-selectin glycoprotein ligand-1 (PSGL-1; *Q* = 4.0 × 10^−7^), tPA (*Q* = 4.0 × 10^−7^), and the Fas receptor (FAS; *Q* = 4.9 × 10^−7^). Clustering was performed (Figure 2B) and revealed that the 109 proteins were mainly involved in immune response, apoptotic processes, blood coagulation, and lipid metabolism (term expansions of all proteins are available in eAppendix 1 in Supplement 3).

### AA/DHA Supplementation and Neonatal Protein Profile

After FDR, we found no association with protein profiles and AA/DHA supplementation (eTable 2 in Supplement 4).

### PNA-Dependent Protein Profiles and Infants Developing Severe ROP

**Figure 3.**
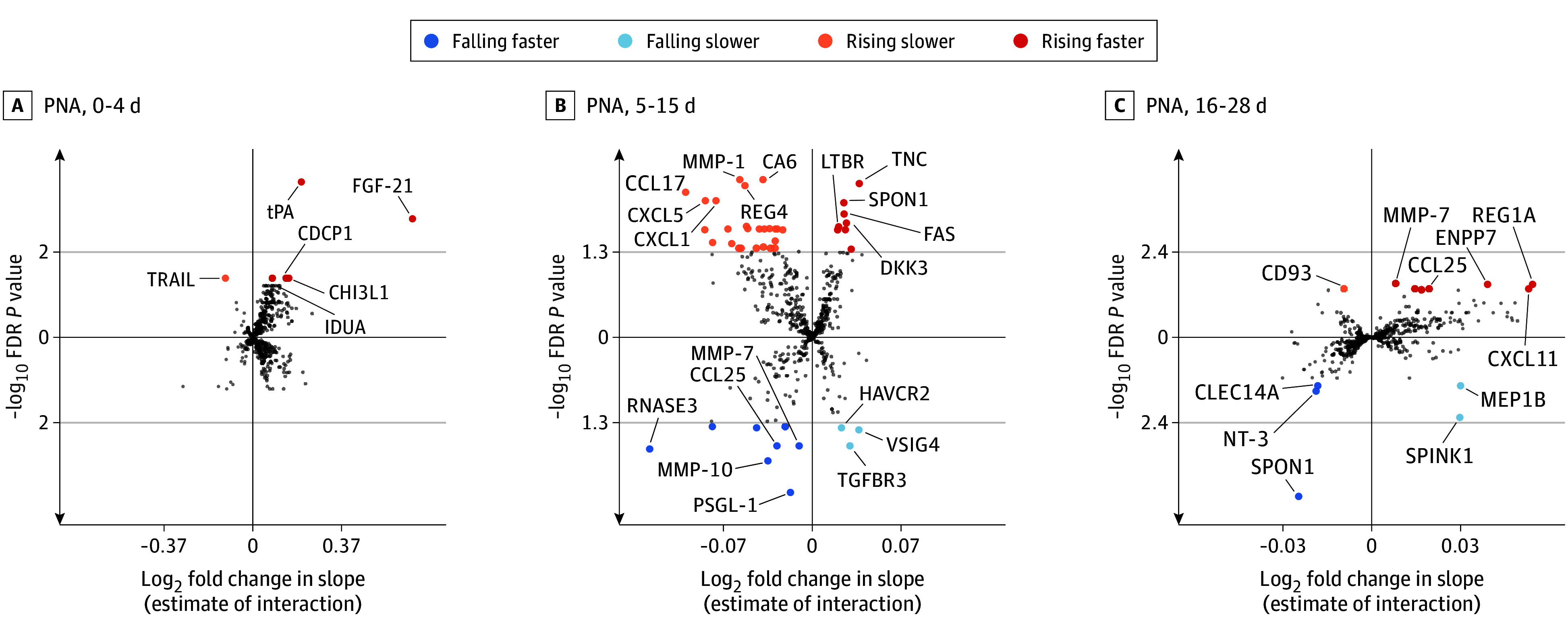
Results From Piecewise Mixed Models for Repeated Measures Comparing No Severe Retinopathy of Prematurity (ROP) and Severe ROP Scatterplots showing log_2_ fold-change in slope against −log_10_ false discover rate (FDR) corrected *P* value in infants with severe ROP vs no severe ROP, where the upper left quadrant represent proteins rising slower (negative β-estimate and increasing levels over postnatal age [PNA]), upper right quadrant proteins rising faster (positive β-estimate and increasing levels over PNA), lower left quadrant falling faster (negative β-estimate and decreasing levels over PNA), and lower right quadrant falling slower (positive β-estimate and decreasing levels over PNA), in PNA days A, 0-4; B, 5-15; C, 16-28. Term expansions of all proteins are available in eAppendix 2 in Supplement 3.

The piecewise linear models identified after FDR—6, 44, and 13 proteins in the first, second, and third interval, respectively—differed between infants with and without severe ROP (eTable 3 in Supplement 4). In total, we identified 58 protein profiles rising faster, rising slower, falling faster, or falling slower in relation to severe ROP (Figure 3A-C) (term expansions of all proteins are available in eAppendix 2 in Supplement 3). Protein examples selected by their fold-change at different periods are shown in eFigure 3A-D in Supplement 3. The protein with the largest effect size was FGF-21, (β = 0.68; 95% CI, 0.39-0.97; *Q *=.002), and tPA presented the second-largest effect size (β = 0.21; 95% CI, 0.13-0.29; *Q *<.001), both with a faster rise from PNA day 0 to 4 in severe ROP cases (eTable 3 in Supplement 4). Functional annotation of proteins differing in infants with severe ROP in PNA days 0 to 4 showed involvement in catabolic processes, intracellular signaling, and in the fibrinolytic system (eFigure 3E in Supplement 3). Proteins differing depending on ROP status during PNA days 5 to 15 (eFigure 3F in Supplement 3) and PNA days 16 to 28 (eFigure 3G in Supplement 3) were primarily involved in inflammation and immune responses.

### Variables and Changes in FGF-21 and t-PA levels

**Figure 4.**
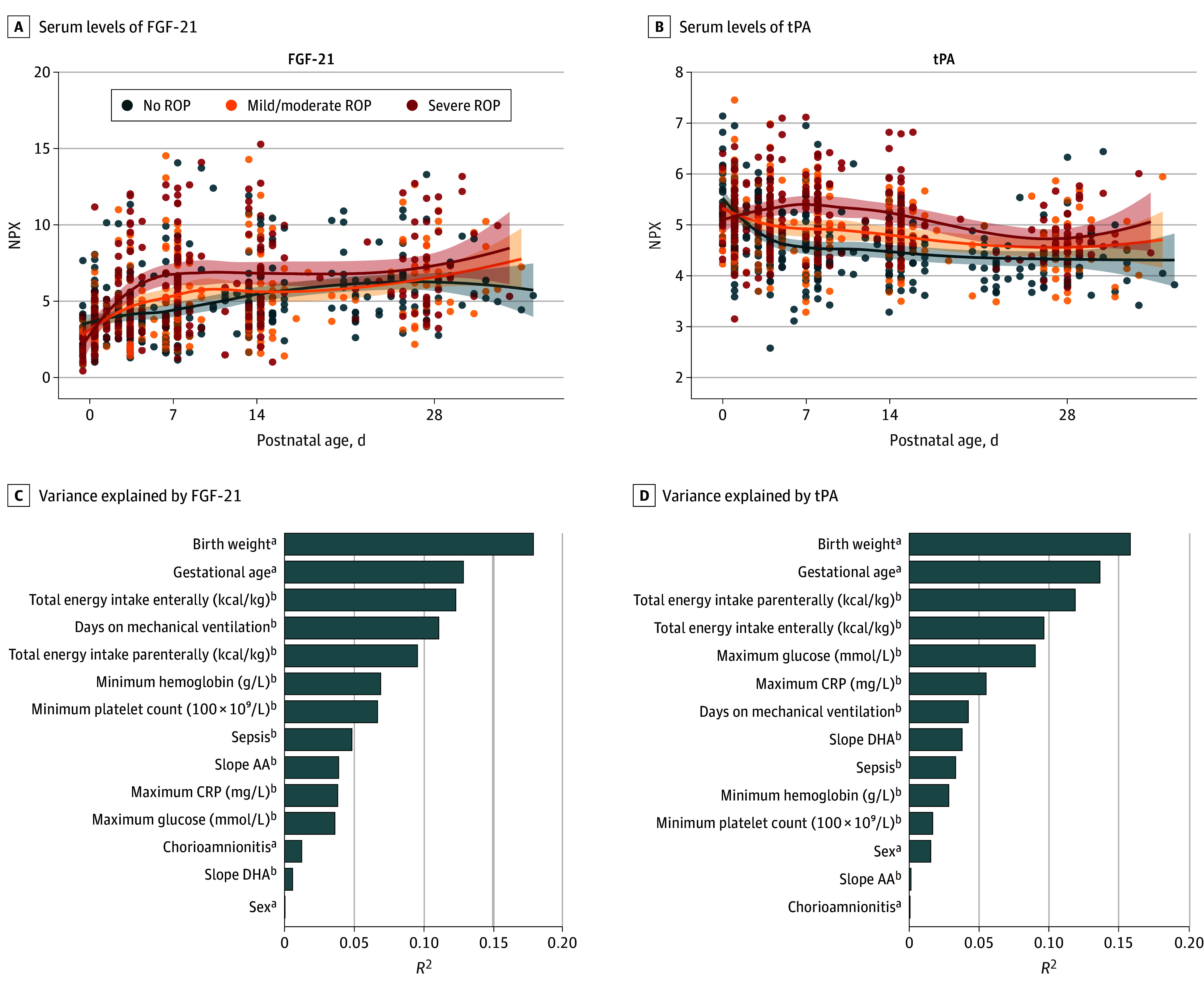
Longitudinal Changes in Fibroblast Growth Factor 21 (FGF-21) and Tissue Plasminogen Activator (tPA) and Associations With Clinical Variables A and B, Serum levels of FGF-21 and tPA across postnatal age (days), stratified by final retinopathy of prematurity (ROP) stage: no ROP, mild/moderate ROP (stages 1-2), and severe ROP (stage 3 and/or treated). C and D, Variance explained (*R*^2^) from univariable linear regression models for the change in FGF-21 and tPA during the first postnatal week by selected clinical variables. ^a^Birth characteristics. ^b^Events or measures during the first postnatal week.

Plotting FGF-21 and tPA levels during the first month of life according to ROP stage revealed that levels in the first PNA week showed the largest differences with respect to ROP outcome (Figure 4), confirming the results from the mixed model for repeated measures analysis. The most prominent contributors to the variability in the FGF-21 slope were birth weight, GA, amount of enteral energy, and days on mechanical ventilation in the first week of life. Variance in t-PA slope was explained mostly by GA, birth weight, and the amount of enteral and parenteral nutrition (Figure 4, eTable 4 in Supplement 4, and eFigure 4 in Supplement 3).

## Discussion

In this post hoc exploratory analysis of data from the Swedish MDM RCT of extremely preterm infants comparing additional enteral postnatal supplementation with AA and DHA and standard nutrition, we analyzed the serum proteome of 177 extremely preterm infants. We found that during the first month of life, proteins associated with the immune system, apoptosis, blood coagulation, and lipid metabolism—such as PSGL-1, t-PA, and FAS—were associated with later severe ROP. Our findings confirmed associations between severe ROP and levels of proteins reported earlier, such as IGF-binding protein 1 (IGFBP-1),^14^ matrix metalloproteinase proteins (MMP) like MMP-1 and MMP-9,^15,16^ and growth hormone.^17^

When evaluating the timing and direction of protein changes, we found that the metabolic stress-induced hormone FGF-21 showed the most pronounced effect size, with a faster rise during the first days of life in infants who later developed severe ROP. Previous reports on the involvement of FGF-21 in the pathogenesis and potential treatment of ROP primarily derive from preclinical models.^18,19,20^ tPA, a crucial component of the fibrinolytic system, showed the second most pronounced effect size for severe ROP, also rising faster in the first postnatal days. To our knowledge, tPA has not been previously reported to be associated with ROP. The functional annotation of proteins associated with severe ROP identified in the first postnatal days revealed their roles in catabolic processes, intracellular signaling, and the fibrinolytic system. Thereafter, proteins involved in inflammatory and immune responses became more prominent, a phenomenon previously described in ROP development.^21,22^ The pronounced early rise in FGF-21, occurring weeks before ROP detection, is consistent with the 2-phase pathophysiology of the disease, whereby events in early life predispose to the later development of severe ROP.

The fact that AA/DHA supplementation was not associated with changes in blood protein profiles does not exclude the possibility of a localized effect within the retina or other tissues.

### FGF-21

FGF-21 is an endocrine hormone primarily produced by the liver, that responds to metabolic and environmental stress by regulating gluconeogenesis, fatty acid oxidation, and ketogenesis.^23,24^ FGF-21 also provides protection against mitochondrial and oxidative stress, exhibits anti-inflammatory properties, and acts in thrombotic homeostasis.^25,26,27^ In preterm infants, high postnatal levels of FGF-21 are associated with postnatal growth failure due to weakened growth hormone receptor signaling and low IGF-1 expression.^28^ Low serum IGF-1 level and poor weight gain are known risk factors for ROP.^29,30^ Elevated levels of FGF-21 have also been observed in children receiving prolonged parenteral nutrition after small intestine resection, aligning with our results in preterm infants.^31^ FGF-21 blood levels are increased during systemic inflammation, including sepsis in neonates,^32^ and levels correlate with sepsis severity and mortality in critically ill adults.^33^ Clinically, FGF-21 is a promising biomarker for mitochondrial diseases in children.^34^

The bioenergetic failure of extremely preterm infants is multifactorial. They have limited fat stores, lose their maternal nutrient supply, enteral feeding is usually not well tolerated, and parenteral nutrition fails to replicate the complex maternal nutritional components.^35^ Additionally, infections, immature antioxidant capacity, and high and fluctuating oxygen delivery can contribute to oxidative stress.^36^

### FGF-21 and ROP

FGF-21 has been linked to ROP mainly through experimental studies using the mouse model of oxygen-induced retinopathy (phase I and II ROP) as well as the mouse model of hyperglycemia-associated retinopathy (phase I ROP).^18,19,20^ In mouse hyperglycemia-associated ROP, FGF-21 administration promoted physiological retinal vessel growth through mitochondrial lipid oxidation mediated by adiponectin.^19^ In preterm infants, low serum adiponectin levels, linked to insufficient DHA levels, have been identified as a risk factor for ROP.^37^

Our results suggest that the variability in FGF-21 levels during the first week of life associated with the degree of infant immaturity, low amount of enteral energy intake, and prolonged duration on mechanical ventilation. The impact of immaturity on rising FGF-21 levels is expected, as the most premature infants are the least equipped to handle metabolic and oxidative stress, which contributes to neonatal morbidities such as ROP.^36,38,39,40,41^ The impact of low enteral nutrition on FGF-21 likely reflects the infant’s degree of bioenergetic failure and increased parenteral nutrition, a key factor in ROP development.^42,43^ The protective effects of sufficient energy intake, early enteral nutrition (specifically breast milk), and fatty acid supplementation against neonatal morbidities such as ROP have been extensively described.^4,5,44,45^ The duration and need for mechanical ventilation are strongly associated with increased stress responses and systemic inflammation in premature infants.^46^

### tPA and MMPs

The second-largest effect size on severe ROP development was found for t-PA, a serine protease that breaks down blood clots, modulates inflammation, and stimulates VEGF expression and angiogenesis.^47^ In preterm infants, elevated tPA levels at birth are associated with infant respiratory distress syndrome and severe illness.^48,49^ To our knowledge, tPA has only indirectly been associated with ROP before, as MMP-9, also found to associate with ROP, is activated by tPA and stimulates endothelial cell migration to the circulation and enhances angiogenesis.^50^ We have previously reported that higher levels of tPA associate with thrombocytopenia, and with lower levels of AA and DHA.^7^ Thrombocytopenia is an established risk factor for ROP.^51,52^ The variables primarily affecting tPA were infant immaturity and enteral and parenteral energy intake during the first week of life, again enhancing the complicated but importance of bioenergetic balance in the youngest infants.

### Comparison With Other Proteome Studies

In the present study, longitudinal protein analysis was performed during the first weeks of life, corresponding to the initial phase of ROP. Other studies have examined early postnatal protein levels in relation to ROP and, eg, reported higher levels of fibroblast growth factor 19 (FGF-19),^53^ myeloperoxidase (MPO),^22^ interleukin 8 (IL-8),^22^ interleukin 6 (IL-6),^54^ ERBB2 (formerly human epidermal growth factor receptor 2 [HER2]),^55^ and galanin (GAL),^55^ in infants who later developed ROP. Conversely, lower levels of mitochondrial superoxide dismutase,^53^ angiopoietin 1 (ANGPT1),^56^ IL-17 levels,^54^ and tumor necrosis factor receptor superfamily member 4 (TNFRSF4)^55^ have also been reported in ROP infants. Several of these proteins (ANGPT1, FGF-19, GAL, IL-6, IL-8, IL-17, MPO) were included in our analyses but were not found to differ between groups. This discrepancy may be attributed to differences in sample timing, proteomics platforms, or the definition of ROP outcomes.

By analyzing proteomics profiles over time rather than at isolated time points, our statistical approach may help clarify the complex dynamics of proteins associated with ROP.

### Limitations

The post hoc exploratory design of this analysis does not permit causal inference regarding the relationship between the identified proteins and ROP. The selected targeted protein panels represent a limited and biased subset of the full circulatory proteome, as our panels were enriched for inflammation- and immune-related proteins. Validation of the identified proteins and pathways in independent cohorts, along with broader proteomics approaches with greater coverage, may offer deeper insights into the pathophysiology of ROP.

## Conclusions

Early proteomic profiles in extremely preterm infants may reveal modifiable processes in the early stages of severe ROP. The association of an early rise in FGF-21 level in this post hoc analysis of the MDM RCT suggests that bioenergetic stress and energy deficiency may be factors in the first phase of ROP. We hypothesize that improved energetics with more maternal milk rich in nutrients and AA/DHA supplementation may reduce the ROP burden. Further studies are needed to validate the changes in FGF-21 levels and determine its relevance as a biomarker for severe ROP. The roles of tPA and possibly MMPs in ROP are poorly known and probably warrant further studies.

## Notes

Supplement 1.
Trial Protocol.

Supplement 2.
Statistical Analysis Plan.

Supplement 3. **eFigure 1.** Flow Diagram of the Study Population in the Primary Randomized Controlled Trial **eFigure 2.** Graphical Representation of the Cohort and Study Design **eAppendix 1.** Figure 2 Protein Expansions **eAppendix 2.** Figure 3 Protein Expansions **eFigure 3.** Results From Piecewise Mixed Models for Repeated Measures Comparing No Severe ROP and Severe ROP **eFigure 4.** Graphic Presentation of the Association Between Infant Characteristics and Selected Variables Present in the First Week of Life and FGF-21 and tPA

Supplement 4. **eTable 1.** Cubic Spline Model Comparing Protein Levels Over Postnatal Age in No/Mild/Moderate ROP vs Severe ROP **eTable 2.** Cubic Spline Model Comparing Protein Levels Over Postnatal Age in Standard Care vs AA/DHA Supplementation **eTable 3.** Piecewise Spline Model Comparing Protein Levels Over Postnatal Age in No/Mild/Moderate ROP vs Severe ROP **eTable 4.** Explained Variances for Early Changes in FGF-21 and tPA, Using Univariate Logistic Regression

Supplement 5.
Data Sharing Statement.
