# Supplementary material for: Proteomic Profile in Retinopathy of Prematurity: A Secondary Analysis of the Mega Donna Mega Randomized Clinical Trial
Source: JAMA Ophthalmol. 2026 Jan 8;144(2):174–84. doi: 10.1001/jamaophthalmol.2025.5594 (PMC12784271; doi:10.1001/jamaophthalmol.2025.5594)
Supplement: Supplement 2. — Statistical Analysis Plan. [file jamaophthalmol-e255594-s002.pdf]

Statistical Analysis Plan

FINAL

Mega Donna Mega

A Randomized Intervention, Multi-Center Study to Determine the Role of Fatty Acids in Serum in preventing Retinopathy of Prematurity

20191119

Author

Name/Title:	
Aldina Pivodic / Statistician	
....	....
Signature:	Date

Approvals

Name/Title:	
Ann Hellström / Principal Investigator	
....	....
Signature:	Date
Name/Title:	
Nils-Gunnar Pehrsson / Independent Statistician	
....	....
Signature:	Date
Name/Title:	
David Ley / Investigator	
....	....
Signature:	Date

Revisions

Version	Description of Changes	Date

Table of Contents

1 Study Details 8
1.1 Study Objectives 8
1.2 Study Design 8
1.3 Treatment Groups 11
1.4 Sample Size 11
2 Study Populations 11
2.1 Definition of Study Populations 11
2.1.1 Intention-To-Treat Population 11
2.1.2 Per-Protocol Population 11
2.1.3 Safety Population 12
3 Study Variables 12
3.1 Baseline Variables 12
3.1.1 Demographics and Baseline Characteristics 12
3.1.2 Prior and Concomitant Medications 13
3.2 Efficacy Variables 13
3.2.1 Primary Efficacy Variable 13
3.2.2 Secondary Efficacy Variables 13
3.2.3 Exploratory Efficacy Variables 14
3.3 Safety Variables 14
3.3.1 Exposure and Compliance of Study Drug 14
3.3.2 Adverse Events 14
4 Statistical Methodology 16
4.1 General Methodology 16
4.2 Patient Disposition and Data Sets Analyzed 17
4.3 Protocol Violations/Deviations 17
4.4 Demographics and Baseline Characteristics 17
4.5 Prior and Concomitant Medications 17
4.6 Efficacy Analyses 17
4.6.1 Primary Efficacy Analysis 17
4.6.2 Secondary Efficacy Analyses 18
4.6.3 Exploratory Efficacy Analyses 19
4.7 Safety Analyses 19
4.7.1 Exposure of Study Drug 19
4.7.2 Adverse Events 19
5 Interim Analyses 19

6 Changes of Analysis from Protocol 20
7 Listing of TableS AND Figures 20
7.1 Listing of Tables 20
7.2 Listing of Figures 20

LIST OF ABBREVIATIONS

**Abbreviation**	**Definition**
AE	Adverse Events
AIC	Akaike's Information Criterion
ASA	American Statistical Association
BPD	Bronchopulmonary Displasia
BL	Birth Length
BW	Birth Weight
CI	Confidence Interval
DBP	Diastolic Blood Pressure
DTI	Volumetric and Diffusor Tensor Imaging
GA	Gestational age
GDM	Gestational Diabetes Mellitus
HC	Head Circumference
HR	Hazard Ratio
IP	Investigational Product
IQR	Interquartile Range
ITT	Intention-To-Treat
IVH	Cerebral Intraventricular Haemorrhage
MMRM	Mixed Models for Repeated Measures
MRI	Magnetic Resonance Imaging
NEC	Necrotizing Enterocolitis
PDA	Patent Ductus Arteriosus
PMA	Postmenstrual Age
PNA	Postnatal Age

PP	Per Protocol
PT	Preferred Term
ROP	Retinopathy of Prematurity
RR	Relative Risk
SAP	Statistical Analysis Plan
SBP	Systolic Blood Pressure
SD	Standard Deviation
SDS	Standard Deviation Score
SOC	System Organ Class

Document note:

This Statistical Analysis Plan (SAP) is describing the planned statistical analyses to be performed for the MDM study's first publication including study population description, evaluation of primary efficacy analysis, important secondary analyses and summary of adverse events.

The SAP is based on study protocol MEGADONNAMEGA 16-7, version 7b from June 2017, and the study link at https://clinicaltrials.gov.

The statistical analyses planned and that will be included in the first publication are applying the recently published guidelines by the American Statistical Association (ASA) that are currently employed by e.g. NEJM:

1. Ronald L. Wasserstein, Allen L. Schirm & Nicole A. Lazar (2019)
Moving to a World Beyond "$p < 0.05$", The American Statistician, 73:sup1, 1-19, DOI:10.1080/00031305.2019.1583913

2. Ronald L. Wasserstein & Nicole A. Lazar (2016) The ASA Statement on *p*-Values: Context, Process, and Purpose, The American Statistician, 70:2, 129-133, DOI: 10.1080/00031305.2016.1154108

3. Alex Dmitrienko, and Ralph B. D'Agostino, Multiplicity Considerations in Clinical Trials. 2018 N Engl J Med 2018;378:2115-22.

4. David Harrington, Ralph B. D'Agostino, Constantine Gatsonis, Joseph W. Hogan, David J. Hunter, Sharon-Lise T. Normand, Jeffrey M. Drazen, and Mary Beth Hamel. New Guidelines for Statistical Reporting in the Journal. 2019 N Engl J Med 381;3

1 STUDY DETAILS

1.1 Study Objectives

The primary objective in this study is to investigate whether enteral administration of AA and DHA in addition to commonly used regimens with parenteral olive based lipid emulsion (Clinoleic) compared to Clinoleic alone prevents the sight threatening disease Retinopathy of Prematurity (ROP).

The secondary objectives in this study are to evaluate following:

1. Postnatal serum fatty acid composition in preterm infants with and without AA:DHA supplementation
2. Postnatal brain development, as assessed by Magnetic Resonance Imaging (MRI), Volumetric and Diffusor Tensor Imaging (DTI) at 40 weeks postmenstrual age and motor and cognitive development at 2 years corrected age and 5.5 uncorrected age. (*data will be available and analysed at later stage*)
3. Neonatal glucose metabolism (*data will be available and analysed at later stage*)
4. Postnatal growth development (weight, length, head circumference)
5. Frequency of neonatal morbidities such as Bronchopulmonary Displasia (BPD), cerebral Intraventricular Haemorrhage (IVH), Patent Ductus Arteriosus (PDA), sepsis and Necrotizing Enterocolitis (NEC)
6. Postnatal body composition by Pea-Pod (*data will be available and analysed at later stage*)

1.2 Study Design

This study is a randomized intervention, multi-center study to determine the role of fatty Acids in serum and breast milk in preventing ROP. Infants without major malformations born at a gestational age (GA) of <28 weeks +0 days will be included.

Randomization will be as follows; GA ≤ 24 weeks +6 days, n=84, GA 25 weeks +0 days to ≤ 26 weeks +6 days, n=84 and GA 27 weeks +0 days to ≤ 27 weeks +6 days, n=42 (in order to receive equal number of infants with conventional treatment and infants treated with AA:DHA supplementation in relation to morbidity outcome. In order to adjust for center variability each center (n=3) will recruit as follows; GA ≤ 24 weeks +6 days, n=14 (conventional) +14 (treated), GA 25 weeks+0 days to ≤ 26 weeks +6 days, n=14 (conventional) +14 (treated) and GA 27 weeks +0 days to ≤ 27 weeks +6 days, n=7 (conventional) +7 (treated).

Study Flow Chart

	day 0	day 1	day 2	hour 72	day 4	day 5	day 6	day 7	day 14	W 3	W 4	W 5	W 6	w 7	PMA 30+0 +-24h	PMA 31+0 +-24h	PMA 32+0+-24h	PMA 33+0+-24h	PMA 34+0+-24h	PMA 35+0+-24h	PMA 36+0+-24h	PMA 37+0+-24h	PMA 38+0+-24h	PMA 39+0+-24h	PMA 40+0+-24h
**Datum**																									
**Gestational age (weeks+days)**																									
Patient info/consent within 36 h	x																								
Physical exam	x																								
Weight+ Length + Head circumference ± 3d from sampling	x							x	x	x	x	x	x	x	x	x	x	x	x	x	x	x	x	x	x
Registration of HR+MABP every second hour up to day 7 (excel)	x	x	x	x	x	x	x	x																	
Study sample cord blood (2 ml). 0h, 72h, d7, d14 (+- 6 h) every other week until PMA 29w and thereafter at 30, 32, 34, 36, and 40 weeks PMA (0,6 ml), (+- 24 h).	x			X				x	x		x		x		x		x		x		x				x

Blood gas	x																								
Chemlab Bilirubin total and conjugated								x	x								x								x
Breast milk samples d7, w32 (same day as study sample), w40								x									x								x
Ultrasound brain day 1, 72 h, day 7, once between day 21-35 and once between w 32-40<br>Pea-Pod		x		x				x		X<br>d<br>2<br>1-<br>3<br>5							X<br>w32-<br>40								X
Ultrasound heart before Pedea treatment, if not treated at d 7				x				x																	
ROP exam with Retcam + SWEDROP																x		x		x		x		x	
Lung provocation test, +/- 3 days<br>appendix 4																					x				
MRI																									x
Questionnaire (participate in clinical trial/eating habits)								x																	x

1.3 Treatment Groups

A randomized intervention study of 210 infants in total, 105 in the Conventional (Clinoleic) group and 105 in the Treated (Clinoleic + AA:DHA) group.

Enteral supplementation with AA:DHA will start at second enteral feeding after birth and continue once daily to postmenstrual week 40 + 0. The supplementation will be delivered prior to feeding (0.1-1ml, according to dosing scheme, see Appendix A in the protocol). If the infant does not tolerate any enteral feeding, the supplement will be given as long as gastric retention is administered.

The intervention group will receive a daily dose of 100 mg AA/kg/day and 50 mg DHA/kg/day (Formulaid™ 2:1 DSM). Dose adjustment will be performed after the infant has regained its birthweight and weight gain results in an increase of 0.1 ml or more (see protocol Appendix A).

1.4 Sample Size

The sample size text below is provided in the study protocol version 7b.

The mean incidence of any ROP in an age-matched population (22 weeks + 0 days to 27 weeks + 6 days of GA at birth) in Sweden during the years 2008 – 2012 is calculated to 42%. Assuming an alpha of 5%, a power of 80% and a 50% reduction in incidence of any ROP, i.e. from 42% to 22%, a sample size of 80 subjects per treatment group is required. The reduction in any ROP with AA:DHA supplementation is based on efficacy in previous publications.

A total of 105+105 subjects will be included to compensate for protocol violations (experience gained in pilot study) and drop-outs (e.g. a perinatal death rate of approximately 15% at these gestational ages).

2 STUDY POPULATIONS

2.1 Definition of Study Populations

2.1.1 Intention-To-Treat Population

All randomized subjects, being correctly included according to the inclusion/exclusion criteria, and have been treated will be included in the Intention-To-Treat (ITT) population.

The final definition of patients included in the ITT population will be made at the clean file meeting prior to database lock. Any exclusions will be described in detail in the clean file protocol.

2.1.2 Per-Protocol Population

All randomized subjects with no major protocol violations will be included in the Per Protocol (PP) population. The final decisions regarding the PP population will be taken at the Clean File meeting before the database lock.

Major protocol violations will be at least following, but not limited to those:

- Final ROP stage available

- Compliance to medication will be evaluated for all infants during the treatment period and sub-periods and appropriate cut-off will be applied. The final decision will be made at the clean file meeting before the database lock.

2.1.3 Safety Population

All enrolled subjects who received at least one dose of randomized IP will be included in the safety population.

3 STUDY VARIABLES

3.1 Baseline Variables

3.1.1 Demographics and Baseline Characteristics

Following variables at birth will be summarized for the infant and analysed per treatment group:

- GA
- Sex
- Birth weight (BW) (gram)
- BW standard deviation score (BWSDS) [for infants born GA>=24 weeks using reference from Niklasson and Albertsson-Wikland 2008]
- Birth length (BL) (cm)
- BL standard deviation score (BLSDS) [for infants born GA>=24 weeks using reference from Niklasson and Albertsson-Wikland 2008]
- Head Circumference (HC) (cm)
- HC standard deviation score (HCSDS) [for infants born GA>=24 weeks using reference from Niklasson and Albertsson-Wikland 2008]
- Twin (yes/no)
- Center (Stockholm, Gothenburg, Lund)

Following variables will be summarized for the mother and analysed per treatment group:

- Age (years)
- Parity (number of child in order)
- Mode of delivery
- Diabetes (type 1, 2 or Gestational Diabetes Mellitus [GDM]) (yes/no)
- Preeclampsia (yes/no)
- Other relevant comorbidity (yes/no,specify)

3.1.2 Prior and Concomitant Medications

Medication taken during the pregnancy by the mother will be summarized per treatment group. Given steroids to the infant during the study will be summarized per treatment group. Other medications will be summarized at later stage in another manuscript.

3.2 Efficacy Variables

3.2.1 Primary Efficacy Variable

Primary efficacy variable in this study is occurrence of sight threatening ROP (ROP stage 3 or worse). All randomized infants will be included in the evaluation of the primary variable. Early drop-outs and deaths before evaluated ROP stage 3 or worse will be considered as non-events and will contribute with their follow-up time in the analysis of comparisons between event rates using Poisson regression. A sensitivity analysis will analyse primary efficacy variable applying survival analyses adjusting for death as competing risk and censoring of those infants that have dropped out due to other reasons than death.

In this study ophthalmologic assessment of ROP stage was performed in blinded manner. Retinal examination were performed approximately once weekly starting at four to five weeks of age according to a standardized protocol and to clinical screening praxis. The evaluation of ROP occurred independently from the study and paediatric ophthalmologists were unaware of which infants were participating in the study.

3.2.2 Secondary Efficacy Variables

Following secondary efficacy variables will be analysed:

- Postnatal serum fatty acid (in mol%) composition in preterm infants with and without AA:DHA supplementation (at 0h, 72h, day 7, day 14, every second week until PMA of 29 weeks and thereafter at 30, 32, 34, 36, 40 weeks PMA). Missing data will not be imputed, analysis will be performed using Mixed Models for Repeated Measures (MMRM) where missing data at random is assumed.
- Outcome of neonatal morbidities (reported as Adverse Events [AE] from birth to 40 weeks PMA)
    - BPD – this morbidity is evaluated at 36 weeks PMA and will be analysed including the complete ITT population with deaths considered as non-events but their follow-up time will contribute to the total follow-up in the analyses of event rate performed by Poisson regression. Sensitivity analysis of BPD will be performed handling all deaths as endpoint also beside confirmed BPD. Drop-out due to other reasons will be considered as non-event.
    - IVH grade 0-4 – this morbidity is evaluated at an early stage post-birth, and larger amount of missing data is not expected. Drop-outs with missing IVH will be handled as non-events. Sensitivity analysis of IVH will be performed handling all deaths with missing IVH as worst value of this endpoint (?). Drop-out due to other reasons will be considered as non-event. Analyses will be performed using Mantel-Haenszel Chi-square test.
    - PDA - this morbidity is evaluated at an early stage post-birth, and larger amount of missing data is not expected. Drop-outs with missing PDA will be handled as non-events. Sensitivity analysis of PDA will be performed handling all deaths with missing PDA as event (?). Drop-out due to other

reasons will be considered as non-event. Analyses will be performed using Fisher's Exact test.

- NEC – this morbidity will be analysed in the same way as the primary variable, using Poisson regression in the main and survival analysis in the sensitivity analysis.

- Postnatal growth development of weight, length, head circumference, at day 0, day 7, day 14, postnatal age (PNA) week 3-7, PMA week 30-40. For infants born at GA>=24 weeks SDS will be calculated and summarized per treatment group. Otherwise, for all infants individual growth curves will be created separately by sex and GA week for the two treatment groups. No imputation of missing data is planned.

3.2.3 Exploratory Efficacy Variables

No other efficacy variables are planned to be analysed within this SAP, for the first study publication.

3.3 Safety Variables

3.3.1 Exposure and Compliance of Study Drug

Exposure will be described as per following for the treated group:

- Number of days in study that infants have been exposed to IP
- Mean daily dose of AA mg/kg/day and DHA mg/kg/day. (They are expected to be 100 and 50 mg/kg/day, respectively at the beginning of the study. Dose adjustment were to be performed after the infant has regained its birthweight and weight gain results in an increase of 0.1 ml or more.)

Compliance will be described as percentage of number of days IP received / total number of days in study * 100.

3.3.2 Adverse Events

Significant AEs are collected from birth onwards during the study, as per the table below. AEs specified event and category are available for the summaries.

Table. Classification of AEs

Category	Event	comments	Mild	Moderate	Severe
Respiratory	Respiratory insufficiency,	requiring intubation			x
	Pulmonary haemorrhage	with resp. symptoms			x
	Pneumothorax				x
	Pleural effusion				x
	Apnoea	treated medically	x		

	Chronic lung disease		x	x	x
Circulatory	Persistent ductus arteriousus (PDA)	≥ 7 days of age without treatment	x		
	Persistent ductus arteriousus (PDA)	treated medically (Ibuprofen, Paracetamol, Furosemid)		x	
	Persistent ductus arteriousus (PDA)	treated surgically			x
	Significant symptomatic hemorrhage				x
	Arterial hypotension	requiring treatment	x	x	x
	Bleeding tendency	requiring treatment (tranexamic acid)	x	x	
	Circulatory arrest				x
Infection	Septicemia	With severe clinical symptoms			x
	Septicemia	with mild clinical symptoms		x	
	Suspected septicemia	Blood culture negative	x	x	
					x
	Other infection	viral, cutaneous	x	x	x
Neurological	Subependymal hemorrhage			x	
	Intraventricular hemorrhage grade 2			x	x
	Intraventricular hemorrhage grade 3 and/or parenchymal hemorrhage				x
	Periventricular leukomalacia	cystic lesions as defined by ultrasound/MR			x
	Periventricular echodensities			x	
	Posthemorragic hydrocefalus	requiring treatment			x

Gastro - intestinal	Necrotizing enterocolitis				x
	Bowel perforation				x
Metabolic	Hyperglycemia	fasting p-gl >10mmol/L in 2 samplings		x	
	Hypoglycemia	<2,6 mmol/L		x	
	Hyperbilirubinemia	Requiring phototherapy	x		
ROP	Retinopathy Of Prematurity	Stage 1, 2	x		
	Retinopathy Of Prematurity	Stage 3		x	
	Retinopathy Of Prematurity	Laser/anti-VEGF treatment and stage 4, 5			x
Other	Major congenital malformation				x

4 STATISTICAL METHODOLOGY

4.1 General Methodology

For continuous variables mean, Standard Deviation (SD), median, minimum and maximum will be presented, or median and Interquartile Range (IQR) as applicable, and for categorical variables number and percentage.

The baseline characteristics will be described; inferential statistics will be performed and interpreted descriptively only.

The confirmatory analyses will be only performed for:

1. Primary efficacy variable, impact of IP on severe ROP (comparison of event rates)
2. Secondary efficacy variable, impact of IP on overall levels of AA (mol%) continuously over time
3. Secondary efficacy variable, impact of IP on overall levels of DHA (mol%) continuously over time
4. Secondary efficacy variable, impact of IP on BPD (yes/no at PMA week 36, comparison of event rates)
5. Secondary efficacy variable, impact of IP on IVH (grade 0-4)

For those analyses the difference in the estimates and their 95% Confidence Intervals (CI) will be provided, and tests will be adjusted for multiplicity according to the fix sequential method as per the sequential order above. This methods allows for the significance mass of 0.05 to be inherited to the next variable in order and the testing is stopped when non-significance is achieved.

Missing data will be handled according to the section 3.2.

All other analyses will be considered as exploratory, 95% CI will be provided for the difference between the groups and p-values will be given only for descriptive purpose.

All tests will be two-tailed and confirmed analyses will be conducted at 0.05 level applying fix sequential testing. All analyses will be performed using SAS software version 9.4 (SAS Institute Inc., Cary, NC, USA).

4.2 Patient Disposition and Data Sets Analyzed

The number of infants included in each of the ITT, PP and safety populations will be summarized by treatment group. The number and percentage of subjects randomized and treated will be presented. Subjects who completed the study and subjects who withdrew from study prematurely will also be presented with a breakdown of the reasons for withdrawal by treatment group for the ITT population.

4.3 Protocol Violations/Deviations

Major protocol deviations are those that are considered to have an effect on the analysis. A list of potential major protocol deviations will be generated programmatically from the data captured before the clean file meeting. The clinical monitors of the study will review the list and the finalisation of the major protocol deviations will be done at the clean file meeting.

The number of patients with major protocol deviations will be summarized per treatment group.

4.4 Demographics and Baseline Characteristics

Demographics and baseline characteristics will be summarized by treatment group for the ITT and PP populations and analyzed according to the methods described in section “General Methodology” above.

4.5 Prior and Concomitant Medications

Prior and concomitant medication will be summarized by higher level group (cortisone, inotropes, insulin, caffeine citrate, diuretics, and all medications linked to AEs, e.g. antibiotics) and generic term by treatment group for ITT population.

4.6 Efficacy Analyses

4.6.1 Primary Efficacy Analysis

The primary analysis in this study is incidence of sight-threatening (severe) ROP, i.e. ROP stage 3 or worse. Due to early deaths and early discontinuations in study the primary analysis will be performed using Poisson regression studying event rates, where deaths and early drop-outs are contributing as non-events with their follow-up time in the study. From this analysis Relative Risks (RR) and 95% CI for comparison between the groups will be presented along with the p-value. The primary analysis will be adjusted for stratification used in the randomization, i.e. adjusted for centre and GA categories as fixed effects, according to the guidelines issued by EMA (*Points to consider on adjustment for baseline covariates*).

The main analysis will be performed on ITT population. The sensitivity analysis will be performed on ITT population comparing cumulative incidence rates adjusted for death as

competing risk, other drop-outs handled as censored in the survival analysis. Gray's test will be performed for this purpose. Moreover, the effect of the sensitivity analysis will be explained by Hazard Ratios (HR) with 95% CI obtained from Cox proportional hazards models. The primary analysis will also be performed on PP population as robustness analysis.

Graphically, cumulative incidence curves with 95% CI will be presented per treatment group.

The analysis will be considered confirmed if the p-value <0.05.

The impact of IP on primary variable will also be evaluated descriptively by centre and by GA categories, used in stratification of the randomization.

4.6.2 Secondary Efficacy Analyses

Confirmatory analyses of secondary variables:

1. The secondary efficacy analyses of fatty acid levels over time will be evaluated by applying MMRM with the change in level of fatty acids (AA and DHA respectively) from birth as dependent variable, and visit, treatment group as fixed effects, fatty acid at birth as fix adjustment variable, with repeated measures by visit applying the covariance pattern (compound symmetry, autoregressive, Toeplitz, unstructured, overall and applied by treatment group) that minimizes the Akaike's Information Criterion (AIC). From this model without an interaction term the overall effect of the IP will be retrieved, presented by adjusted means with 95% CI. The assumption of normally distributed residuals will be checked by reviewing the diagnostic plots. If not satisfactory the sandwich estimators for the standard errors (resulting in wider CIs) will be applied. Additional model for descriptive (exploratory) purpose will be performed including interaction term from which the impact of IP over time will be retrieved and presented graphically.
2. The secondary efficacy analysis of the impact of IP on BPD will be analyzed on ITT population including infants for which BPD was evaluated at 36 weeks PMA, deaths and other drop-outs will be handled as non-events. Poisson regression with log-link function, will be performed, resulting in RRs with 95% CI and associated p-value.
3. The secondary efficacy analysis of the impact of IP on IVH (grade 0-4) will be analyzed on ITT population using all infants that have evaluated IVH, missing data will be considered as non-events. Mantel-Haenszel Chi-square test will be performed.

Following secondary variables will be analyzed descriptively:

1. PDA will be analyzed using Fisher's Exact test. Proportion of patients with events and 95% CI will be provided.
2. NEC will be analyzed in the same way as the primary efficacy variable, applying Poisson regression. RR with 95% CI will be provided.
3. Growth development, by computing difference with 95% CI between treatment groups in BWSDS, BLSDS, HCSDS for studied time points, only on infants born GA>=24 weeks. Individual growth curves will be created by GA week and sex, per treatment group and mean curve estimated per each subgroup of infants.

Main analyses will be performed on ITT population and robustness analyses on PP population. Sensitivity analyses performed on ITT population handling deaths and early drop-outs as per the section 3.2 will also be performed.

4.6.3 Exploratory Efficacy Analyses

No analyses of exploratory variables will be performed.

4.7 Safety Analyses

4.7.1 Exposure of Study Drug

IP exposure and compliance will be summarized for safety population.

4.7.2 Adverse Events

AEs will be summarized for safety population.

A summary of subjects reporting at least one of the following AEs will be presented in an overview table:

- Any AE
- Any SAE
- Any treatment-related AE
- Any treatment-related SAE
- Any AE leading to discontinuation
- Death

Summaries per SOC and PT presenting n (%) of AEs and n (%) of subjects with at least one AE will be provided for:

- All AEs (includes all serious and non-serious AEs)
- All AEs by maximum reported intensity
- All AEs by causality
- All SAEs
- All AEs leading to discontinuation

5 INTERIM ANALYSES

After the first 10 subjects have been treated, pharmacokinetic analyses were performed with respect to fatty acid concentrations in serum.

After the first 30 subjects have been treated and evaluated a safety data committee performed an evaluation to confirm that the safety profile is acceptable and that the assumption of a reduction in ROP incidence is reasonable.

6 CHANGES OF ANALYSIS FROM PROTOCOL

No changes to protocol version 7b have been done. However, no detailed description of the analyses were available in the protocol. All analyses specified within this SAP are aimed to be conservative regarding the IP and applying ICH and EMA guidelines for statistical analyses and adjustments. Additionally, recently published guidelines from ASA and NEJM have been employed.

7 LISTING OF TABLES AND FIGURES

7.1 Listing of Tables

Table Number	Table Title
14.1.1	Patient Disposition and Data Sets Analyzed (ITT Population)
14.1.2	Protocol Deviations Leading to Exclusion from PP Population (ITT Population)
14.1.3.1	Demographics and Baseline Characteristics (ITT Population)
14.1.3.2	Demographics and Baseline Characteristics (PP Population)
14.1.6.1	Prior Medications (ITT population)
14.1.6.2	Concomitant Medications (ITT population)
14.2.1.1	Primary Efficacy Analysis (ITT Population)
14.2.1.2	Analysis of Primary Efficacy Variable (PP Population)
14.2.x	*Other efficacy variables/analyses*
14.2.x	*Exploratory Analysis – xxx*
14.3.1.1	Duration of Exposure and Compliance (Safety Population)
14.3.2.1	Summary of Adverse Events (Safety Population)
14.3.2.2	Adverse Events, by System Organ Class and Preferred Term (Safety Population)
14.3.2.3	Adverse Events, by System Organ Class, Preferred Term and Maximum Reported Intensity (Safety Population)
14.3.2.4	Adverse Events, by System Organ Class, Preferred Term and Causality Assessment (Safety Population)
14.3.2.5	Serious Adverse Events, by System Organ Class and Preferred Term (Safety Population)
14.3.3	Adverse Events Leading to Discontinuation, by System Organ Class and Preferred Term (Safety Population)

7.2 Listing of Figures

Figure Number	Figure Title
14.2.1.1	Cumulative Incidence Curve for Severe ROP by Treatment Group (ITT Population)
14.2.1.2	Cumulative Incidence Curve for Severe ROP by Treatment Group (PP Population)
14.2.2.1	AA Profile by Treatment Group (ITT Population)
14.2.2.2	AA Profile by Treatment Group (PP Population)
14.2.3.1	DHA Profile by Treatment Group (ITT Population)

14.2.3.2	DHA Profile by Treatment Group (PP Population)
14.2.4.1	BPD, IVH Grade and PDA by Treatment Group (ITT Population)
14.2.4.2	BPD, IVH Grade and PDA by Treatment Group (PP Population)
14.2.5.1	Cumulative Incidence Curve for NEC by Treatment Group (ITT Population)
14.2.5.2	Cumulative Incidence Curve for NEC by Treatment Group (PP Population)
14.2.6.1.x	XXXX Development Curves by Treatment Group (ITT Population)
14.2.6.2.x	XXXX Development Curves by Treatment Group (PP Population)
14.3.1	Summary of Adverse Events (Safety Population)
