# Supplementary material for: Proteomic Profile in Retinopathy of Prematurity: A Secondary Analysis of the Mega Donna Mega Randomized Clinical Trial
Source: JAMA Ophthalmol. 2026 Jan 8;144(2):174–84. doi: 10.1001/jamaophthalmol.2025.5594 (PMC12784271; doi:10.1001/jamaophthalmol.2025.5594)
Supplement: Supplement 3. — eFigure 1. Flow Diagram of the Study Population in the Primary Randomized Controlled Trial eFigure 2. Graphical Representation of the Cohort and Study Design eAppendix 1. Figure 2 Protein Expansions eAppendix 2. Figure 3 Protein Expansions eFigure 3. Results From Piecewise Mixed Models for Repeated Measures Comparing No Severe ROP and Severe ROP eFigure 4. Graphic Presentation of the Association Between Infant Characteristics and Selected Variables Present in the First Week of Life and FGF-21 and tPA [file jamaophthalmol-e255594-s003.pdf]

Supplementary Online Content

Lundgren P, Danielsson H, Panwar MB, et al. Proteomic profile in retinopathy of prematurity: a secondary analysis of the Mega Donna Mega randomized clinical trial. *JAMA Ophthalmol*. Published online January 8, 2026. doi:10.1001/jamaophthalmol.2025.5594

eFigure 1. Flow Diagram of the Study Population in the Primary Randomized Controlled Trial

eFigure 2. Graphical Representation of the Cohort and Study Design

eAppendix 1. Figure 2 Protein Expansions

eAppendix 2. Figure 3 Protein Expansions

eFigure 3. Results From Piecewise Mixed Models for Repeated Measures Comparing No Severe ROP and Severe ROP

eFigure 4. Graphic Presentation of the Association Between Infant Characteristics and Selected Variables Present in the First Week of Life and FGF-21 and tPA

This supplementary material has been provided by the authors to give readers additional information about their work.

© 2026 Lundgren P et al. *JAMA Ophthalmology*.

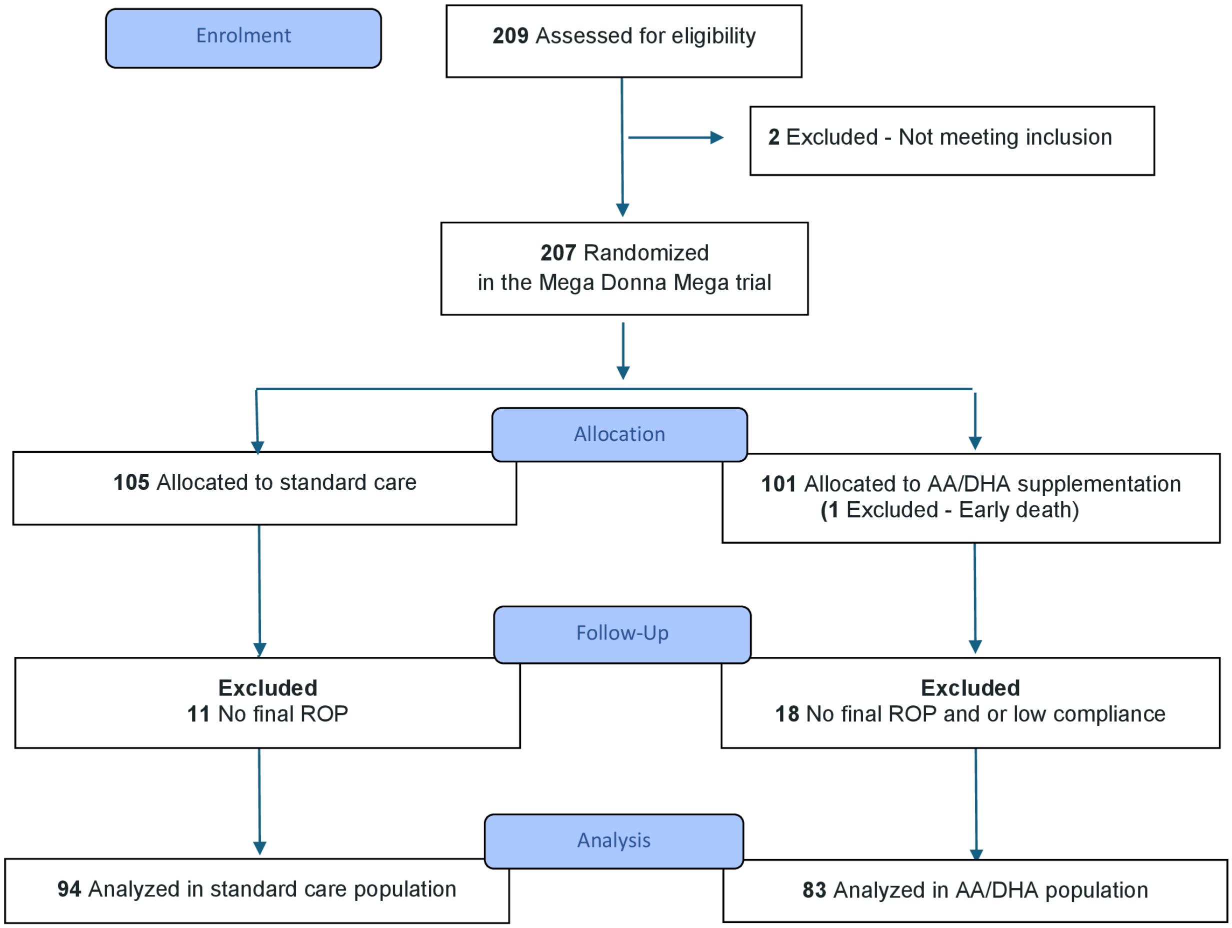

eFigure 1. Flow Diagram of the study population in the primary randomized controlled trial.

© 2026 Lundgren P et al. *JAMA Ophthalmology*.

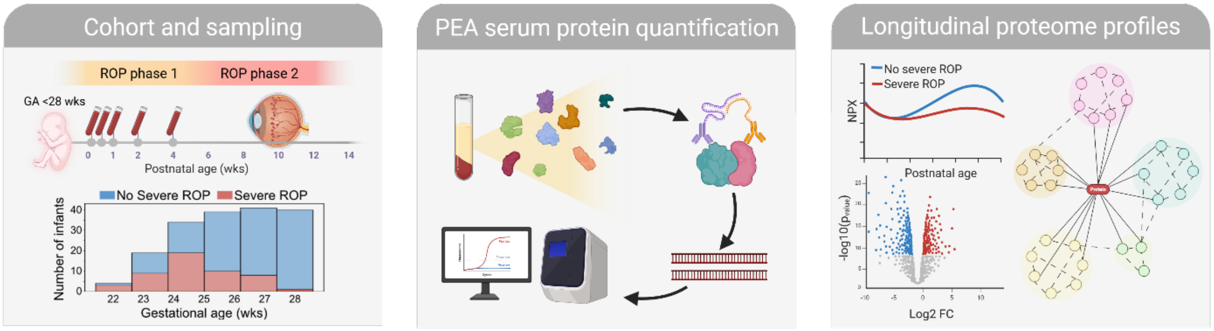

eFigure 2. Graphical representation of the cohort and study design.

© 2026 Lundgren P et al. *JAMA Ophthalmology*.

eAppendix 1. Figure 2 Protein Expansions

ALCAM indicates activated leukocyte cell adhesion molecule; ANGPTL3, angiopoietinlike 3; AOC3, amine oxidase copper containing 3; AP-N, alanyl aminopeptidase membrane; APOM, apolipoprotein M; CA6, carbonic anhydrase 6; CCDC80, coiled-coil domain containing 80; CCL, C-C motif chemokine ligand; CDCP1, CUB domain containing protein 1; CD1C, CD1C molecule; CD6, CD6 molecule; CD93, CD93 molecule; CD209, CD209 molecule; CD300LG, CD300 moleculelike family member G; CHI3L1, chitinase 3–like 1; CHRDL2, chordinlike 2; CLEC14A, C-type lectin domain containing 14A; CLUL1, clusterinlike 1; CNDP1, carnosine dipeptidase 1; COL1A1, collagen type 1 alpha 1 chain; COL18A1, collagen type XVIII alpha 1 chain; CST6, cystatin E/M; CTSD, cathepsin D; CTSL1, cathepsin L; CXCL, C-C-C motif chemokine ligand; DAG1, dystroglycan 1; DKK3, dickkopf Wnt signaling pathway inhibitor; DLK-1, deltalike noncanonical notch ligand 1; DNER, delta/notchlike EGF repeat containing; ENPP7, ectonucleotide pyrophosphatase/phosphodiesterase 7; EPHB4, EPH receptor B4; FABP4, fatty acid binding protein 4; FAS, Fas receptor; FCRL, Fc receptorlike; FDR, false discovery rate; FGF-21, fibroblast growth factor 21; FSTL3, follistatinlike 3; Gal-9, galectin 9; HAVCR2, hepatitis A virus cellular receptor 2; ICAM1, intercellular adhesion molecule 1; IDUA, iduronidase, alpha-L; IGFBP-1, insulinlike growth factor binding protein 1; IGF2R, insulinlike growth factor 2 receptor; IL, interleukin; ITGB2, integrin subunit beta 2; JAM-A, junctional adhesion molecule A; LAIR1, leukocyte associated immunoglobulinlike receptor 1; LDL, low-density lipoprotein; LILRB1, leukocyte immunoglobulinlike receptor B1; LTBR, lymphotoxin beta receptor; MATN2, matrilin 2; MEP1B, meprin A subunit beta; MMP, matrix 12 metalloproteinase protein; MSMB, microseminoprotein beta; NECTIN2, nectin cell adhesion molecule 2; NT-3, neurotrophin 3; PCSK9, proprotein convertase subtilisin/kexin type 9; PDGFRB, platelet-derived growth factor receptor beta; PILRA, paired immunoglobinlike type 2 receptor alpha; PILRB, paired immunoglobinlike type 2 receptor beta; PLC, phospholipase C ; PON3, paraoxonase 3; PSGL-1, P-selectin glycoprotein ligand 1; PSP-D, surfactant protein D; RARRES2, retinoic acid receptor responder 2; REG1A, regenerating family member 1 alpha; REG4, regenerating family member 4; RELT, RELT TNF receptor; RNASE3, ribonuclease A family member 3; SCF, KIT ligand; SCGB3A1, secretoglobin family 3A member 1; SHPS-1, signal regulatory protein alpha; SIRPB1, signal regulatory protein beta 1; SLAMF1, signaling lymphocytic activation molecule family member 1; SPINK1, serine peptidase inhibitor, kazal type 1; SPON1, spondin 1; SSC4D, scavenger receptor cysteine rich family member with 4 domains; TFPI, tissue factor pathway inhibitor; TGFBR3, transforming growth factor beta receptor 3; TIMD4, T-cell immunoglobulin and mucin domain containing 4; TIMP4, TIMP metallopeptidase inhibitor 4; TM, thrombomodulin; TNC, tenascin C; TNF-R1, TNF receptor superfamily member 1A; TNF-R2, TNF receptor superfamily member 1B; TNFB, lymphotoxin alpha; TNFRSF10A, tumor necrosis factor receptor superfamily member 10A; TNFRSF10C, tumor necrosis factor receptor superfamily member 10C; tPA, tissue plasminogen activator; TRAIL, TNF superfamily member 10; TRAIL-R2, TNF receptor superfamily member 10b; U-PAR, plasminogen activator, urokinase receptor; VCAN, versican; VSIG4, V-set and immunoglobulin domain containing 4.

© 2026 Lundgren P et al. *JAMA Ophthalmology*.

eAppendix 2. Figure 3 Protein Expansions

CA6 indicates carbonic anhydrase 6; CCL, C-C motif chemokine ligand; CDCP1,CUB domain containing protein 1; CD93, CD93 molecule; CH13L1, chitinase 3–like 1; CLEC14A, C-type lectin domain containing 14A; CXCL, C-X-C motif chemokine ligand 1; DKK3, dickkopf WNT signaling pathway inhibitor 3; ENPP7, ectonucleotide pyrophosphatase/phosphodiesterase 7; FAS, Fas receptor; FGF-21, fibroblast growth factor 21; HAVCR2, hepatitis A virus cellular receptor 2; IDUA, iduronidase, alpha-L; LTBR, lymphotoxin beta receptor; MEP1B, meprin A subunit beta; MMP, matrix metalloproteinase protein; NT-3, neurotrophin 3; PSGL-1, P-selectin glycoprotein ligand 1; REG4, regenerating family member 4; RNASE3, ribonuclease A family member 3; SPINK1, serine peptidase inhibitor, kazal type 1; SPON1, spondin 1; TGFBR3, transforming growth factor beta receptor 3; TNC, tenascin C; tPA, tissue plasminogen activator; TRAIL, TNF superfamily member 10; VSIG4, V-set and immunoglobulin domain containing 4.

© 2026 Lundgren P et al. *JAMA Ophthalmology*.

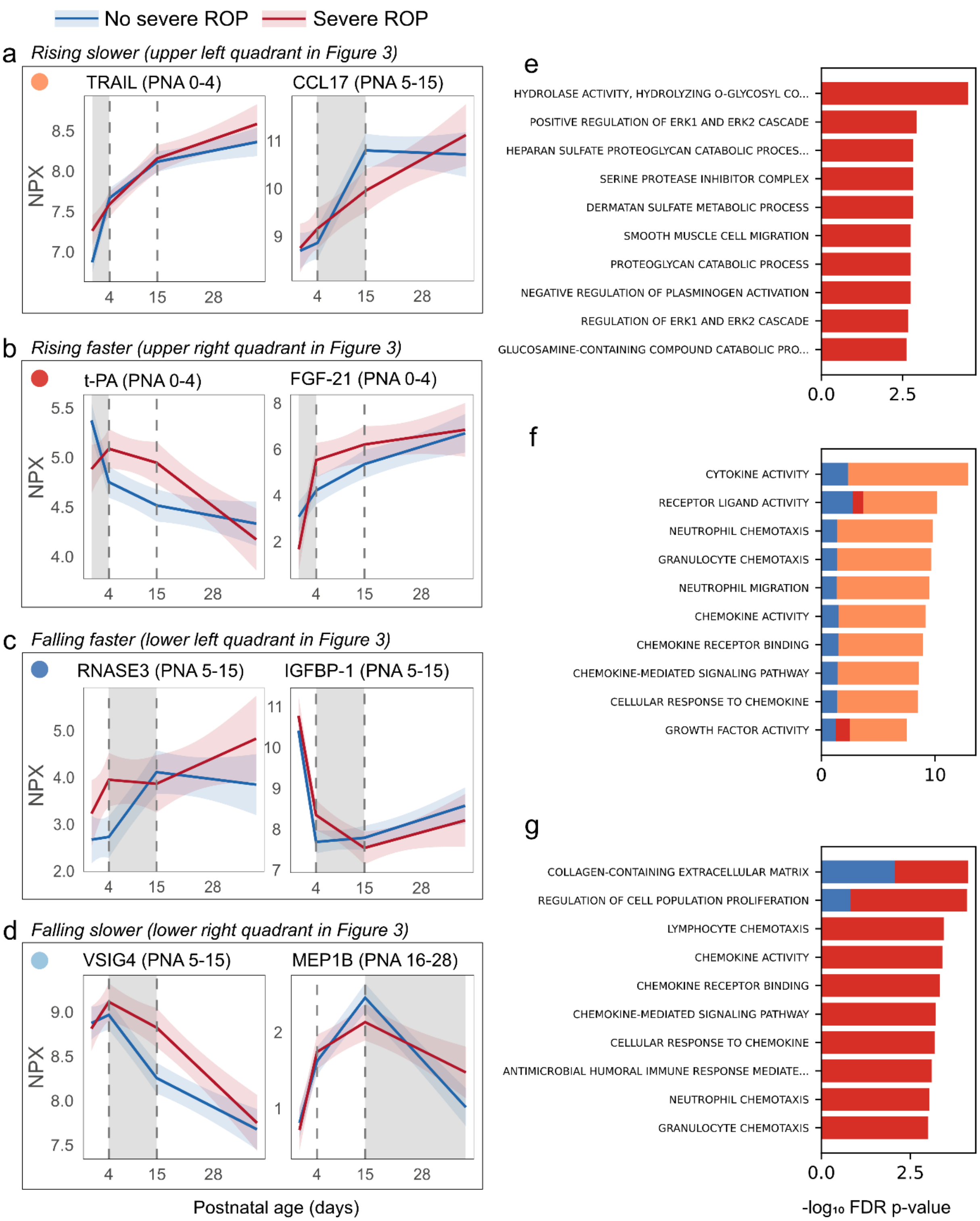

eFigure 3. Results from piecewise mixed models for repeated measures comparing no severe ROP and severe ROP. Examples of model predicted protein trajectories over the first 4 postnatal weeks for proteins:

© 2026 Lundgren P et al. *JAMA Ophthalmology*.

a) Rising slower (negative β-estimate AND increasing levels over PNA, upper left quadrant proteins in Figure 3 scatter plots);
b) Rising faster (positive β-estimate AND increasing levels over PNA, upper right quadrant proteins in Figure 3 scatter plots);
c) Falling faster (negative β-estimate AND decreasing levels over PNA, upper right quadrant proteins in Figure 3 scatter plots);
d) Falling slower (positive β-estimate AND decreasing levels over PNA, lower right quadrant in Figure 3 scatter plots).
The significant period for each protein is highlighted in the shaded gray area.

Bar plots showing over-represented terms (FDR<0.05) from functional enrichment analysis in postnatal days **e**) 0-4; **f**) 5-15; **g**) 16-28. The colors represent the proportional contribution of proteins according to the four categories listed above. PNA; postnatal age.

© 2026 Lundgren P et al. *JAMA Ophthalmology*.

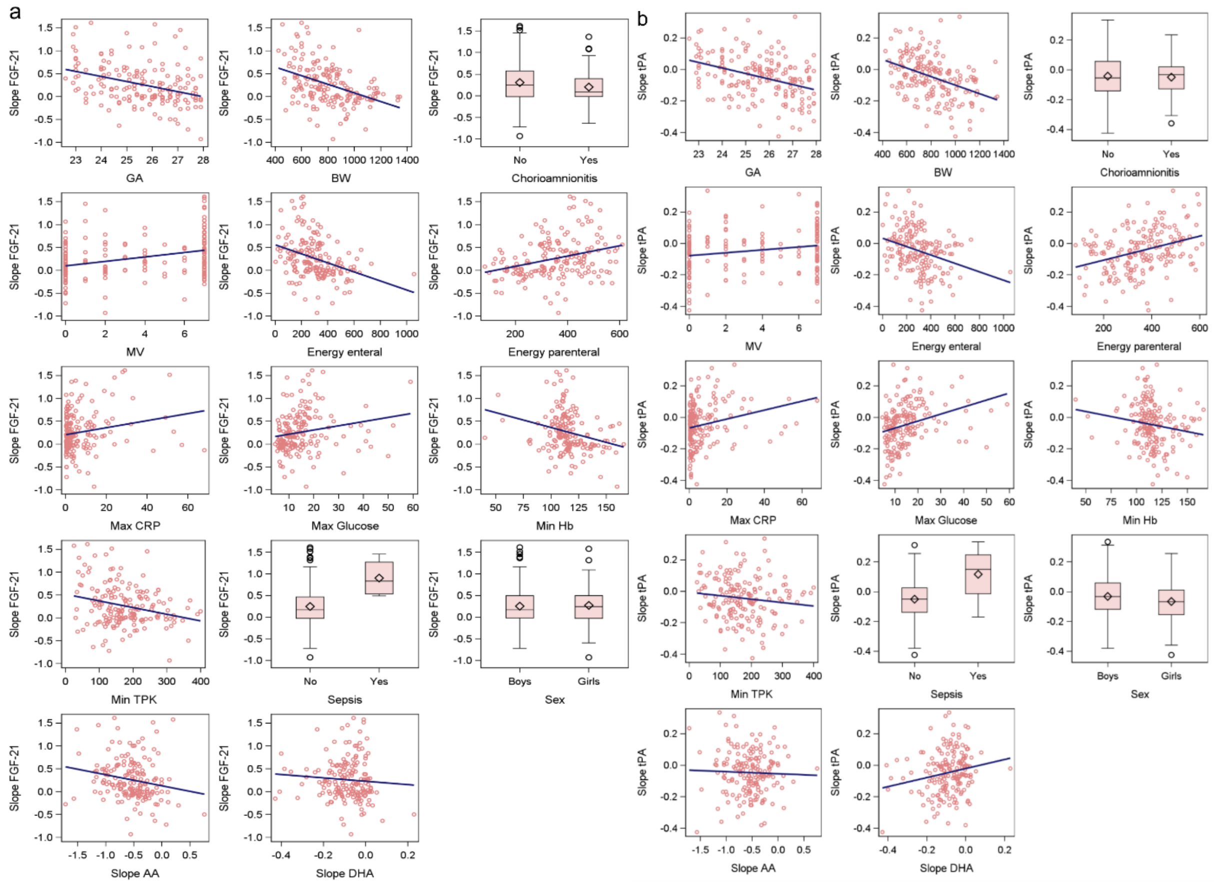

eFigure 4. Graphic presentation of the association between infant characteristics and selected variables present in the first week of life and **a**) FGF-21 and **b**) tPA.

© 2026 Lundgren P et al. *JAMA Ophthalmology*.
