# Supplementary material for: Proteomic Profile in Retinopathy of Prematurity: A Secondary Analysis of the Mega Donna Mega Randomized Clinical Trial
Source: JAMA Ophthalmol. 2026 Jan 8;144(2):174–84. doi: 10.1001/jamaophthalmol.2025.5594 (PMC12784271; doi:10.1001/jamaophthalmol.2025.5594)
Supplement: Supplement 5. — Data Sharing Statement. [file jamaophthalmol-e255594-s005.pdf]

Data Sharing Statement

Lundgren. Proteomic Profile in Retinopathy of Prematurity. *JAMA Ophthalmol*. Published January 08, 2026. doi:10.1001/jamaophthalmol.2025.5594

Data
Additional Information: ClinicalTrials.gov Identifier: NCT03201588
Data available: No

Additional Information
Explanation for why data not available: The datasets generated and/or analyzed during the current study are not publicly available due to ethical permits and The General Data Protection Regulation (GDPR) Regulation (EU) 2016/679 on the protection of natural persons with regard to the processing of personal data and on the free movement of such data law regulates the availability of personal data, but deidentified data are available from the corresponding author on reasonable request.
